# Anti-IL-17A treatment reduces serum inflammatory, angiogenic and tissue remodeling biomarkers accompanied by less synovial high endothelial venules in peripheral spondyloarthritis

**DOI:** 10.1038/s41598-020-78204-6

**Published:** 2020-12-03

**Authors:** Merlijn H. Kaaij, Boy Helder, Leonieke J. J. van Mens, Marleen G. H. van de Sande, Dominique L. P. Baeten, Sander W. Tas

**Affiliations:** 1 Department of Rheumatology and Clinical Immunology, Amsterdam Rheumatology and Immunology Center, Amsterdam UMC, University of Amsterdam, Meibergdreef 9, Amsterdam, The Netherlands; 2 Department of Experimental Immunology, Amsterdam Infection and Immunity Institute, Amsterdam UMC, University of Amsterdam, Meibergdreef 9, Amsterdam, The Netherlands

**Keywords:** Chronic inflammation, Ankylosing spondylitis, Psoriatic arthritis, Rheumatology, Rheumatic diseases, Spondyloarthritis, Immunology, Cytokines, Inflammation

## Abstract

Spondyloarthritis (SpA) is characterized by inflammation and new bone formation. The exact pathophysiology underlying these processes remains elusive. We propose that the extensive neoangiogenesis in SpA could play a role both in sustaining/enhancing inflammation and in new bone formation. While ample data is available on effects of anti-TNF on angiogenesis, effects of IL-17A blockade on serum markers are largely unknown. We aimed to assess the impact of secukinumab (anti-IL-17A) on synovial neoangiogenesis in peripheral SpA, and how this related to changes in inflammatory and tissue remodeling biomarkers. Serum samples from 20 active peripheral SpA patients included in a 12 week open-label trial with secukinumab were analyzed for several markers of angiogenesis and tissue remodeling. Synovial biopsies taken before and after treatment were stained for vascular markers. Serum levels of MMP-3, osteopontin, IL-6 (all *P* < 0.001), IL-31, S100A8, S100A9, Vascular Endothelial Growth Factor A (VEGF-A), IL-33, TNF-α (all *P* < 0.05) decreased significantly upon anti-IL17A treatment. Secukinumab treatment resulted in a decrease in the number of synovial high endothelial venules and lymphoid aggregate score. These results indicate that anti-IL-17A not only diminishes inflammation, but also impacts angiogenesis and tissue remodeling/new bone formation. This may have important implications for disease progression and/or structural damage.

## Introduction

Spondyloarthritis (SpA) is a group of inflammatory diseases that is characterized by inflammation and tissue remodeling in the axial and peripheral skeleton. Tissue remodeling includes new bone formation and typical manifestations include fusion of spine, hip and sacroiliac joints, as well as syndesmophytes in the intervertebral space and enthesophytes at the tendon and ligament insertion sites^1^. New bone formation contributes to disability and impairs function in patients^2^. However, the underlying pathophysiology that contributes to this process is largely unknown. We hypothesize that angiogenesis may play a crucial role in sustaining inflammation and in new bone formation. Angiogenesis is known to be increased in SpA compared to rheumatoid arthritis synovial tissue (ST)^3,4^, which is substantiated by increased ultrasound Doppler signals and elevated VEGF levels that are linked to radiographic progression^1,5^. Of note, angiogenesis is also necessary for bone formation^6^ and recently a vessel subtype that links these processes was described^7^. In mice these so-called type H vessels are involved in the attraction of osteoprogenitors and are characterized by high expression of CD31 and endomucin, whereas in humans CD105/endoglin seems to be a hallmark of these vessels^8^.

TNF inhibition reduces both inflammation and angiogenic responses, and has improved outcome in SpA^9,10^, but whether anti-TNF treatment also halts new bone formation remains to be determined^11^. The introduction of secukinumab, an anti-IL-17A monoclonal antibody, as new therapeutic agent for SpA has shown therapeutic efficacy with reduction of inflammation^12,13^, but detailed information on its impact on angiogenesis or radiographic progression is hitherto very limited^14^. IL-17 is known to contribute to inflammation by affecting fibroblasts, endothelial cells, osteoblasts and osteoclasts, all important cell types in SpA pathogenesis^15^. IL-17A can induce angiogenesis through stimulating secretion of angiogenic factors such as vascular endothelial growth factor (VEGF)^16^, as well as tissue remodeling through factors like matrix metalloproteinases (MMPs) and RANKL^15^. Interestingly, IL-17 is also important in the formation of ectopic lymphoid structures (ELS), which can be present in synovial inflammation as well^17^. IL-17 secreting T cells, such as lymphoid tissue inducer-like cells and Th17 cells, may initiate ELS formation^17^. Large ELS are accompanied by high endothelial venules (HEVs) in arthritis^18^ which may be induced by endothelial cell (EC)-LTβR signaling^19^. LTβR mediated HEV formation likely requires non-canonical NF-κB signaling^20^ and we have demonstrated that this pathway is active in microvessels in the inflamed ST of patients with various types of arthritis^21^.

Anti-TNF and anti-IL-17A therapy are both effective in SpA, but not all patients respond to these treatments^22^. Biomarkers that predict treatment response and effects on tissue remodeling are largely lacking^23^. Detailed knowledge on the mechanisms underlying the beneficial effects of anti-IL-17A treatment will help identifying potential biomarkers to predict treatment response. Furthermore, it is also crucial to better understand why this treatment may not be effective in all (subtypes of) SpA patients. Therefore, the purpose of this study was to identify which pathogenic processes are affected by IL-17A therapy by analyzing several well-known serum biomarkers of inflammation, angiogenesis and tissue remodeling/new bone formation in patients with peripheral SpA (pSpA) treated with secukinumab and by analyzing the effects on angiogenesis and in particular specialized blood vessel subsets in the inflamed synovium.

## Materials and methods

### Study population

For our analysis we used 2 different sets of patient samples. Our main cohort consisted of 20 active pSpA patients that met the Assessment of SpondyloArthritis International Society classification criteria^24^ for pSpA, who participated in a 12 week mechanism of action study within a 116 week open-label, investigator initiated, clinical trial with secukinumab. Secukinumab 300 mg sc was given weekly for the first four weeks, followed by 300 mg sc every four weeks. All 20 pSpA patients from the original study were selected: 13 patients with psoriatic arthritis (PsA), 3 patients with undifferentiated SpA, 2 patients with ankylosing spondylitis with peripheral arthritis, 1 patient with reactive arthritis and 1 patient with inflammatory bowel disease (IBD) associated pSpA (IBD was in remission at screening and baseline). Additional clinical data can be found in the original manuscript^13^. All participants gave written informed consent and this study was approved by the local ethics committee of the Amsterdam Medical Center. All methods were performed in accordance with relevant guidelines and regulations. Serum was obtained and ST samples were taken from an inflamed knee or ankle joint via mini-arthroscopy before and 12 weeks after treatment with secukinumab^25^. Synovial biopsies were snap frozen in Tissue-Tek (Sakura Finetek) for cryosectioning and subsequent staining procedures. Detailed study design, further patient characteristics and primary results were previously published^13^.

To investigate potential differences in synovial inflammation between SpA and RA, we made use of a different set of ST biopsy samples collected cross-sectionally from inflammatory arthritis patients consisting of 13 SpA (11 PsA, 1 pSpA and 1 with undifferentiated SpA) patients and 15 RA patients with active established disease that were biologic naive and underwent an arthroscopy to obtain and process biopsies as mentioned above^25^. Samples were selected based on availability of synovial tissue. Disease activity was determined primarily by history, physical examination and at least one inflamed joint suited for arthroscopy (ankle or knee). All patients gave written informed consent, as approved by the Ethics committee of the Academic Medical Center.

### Luminex Serum assay

Serum levels of cytokines, growth factors and other soluble factors focused on inflammation, angiogenesis and tissue remodeling were measured in all patients in the secukinumab study at baseline and at the 12 week timepoint using the Human Magnetic Luminex Assay (LXSAHM, R&D Systems). Biomarkers were selected based on literature and availability in existing luminex panels. Serum samples were diluted twofold and 50 µL sample or standard was used for the luminex assay in accordance with the manufacturer’s instructions**.** The cytokines BMP-2, CD40 Ligand, DKK-1, CD105 (Endoglin), IL-31, IL-33, IL-6, MMP-3, Osteopontin, ROBO4, S100A8, S100A9, sclerostin (SOST), TIE-2, TNF-α, VCAM-1, Vascular Endothelial Growth Factor A (VEGF-A) were measured.

### Immunohistochemistry

Immunohistochemical stainings were performed with 5-uM cryostat sections. Sections were thawed for 20 min, followed by acetone fixation for 10 min at room temperature (RT), endogenous peroxidase blocked for 30 min and incubated overnight at 4 degrees Centigrade with the primary antibody against CD20 B cells (L26, Dako) or matched isotype control. After PBS washing, sections were incubated with a biotinylated horseradish peroxidase-conjugated streptavidin secondary antibody. Lymphoid aggregates were assessed on anti-CD20 and anti-CD3 stained slides, according to earlier used methods^18,26^. Aggregate scores were determined by counting the number of cells in the radius of the aggregate, classified as grade 1 with a 2–5 cell radius, grade 2 with a 6–10 cell radius or grade 3 with a radius of > 10 cells. Sections without lymphocyte aggregates were scored as 0. Slides were evaluated by two observers (MHK and BH) blinded for the patients identity and moment of biopsies.

### Immunofluorescence

Immunofluorescent (IF) stainings were also performed with 5-uM cryostat sections and acetone fixed for 10 min at RT. Sections were dried for 10 min, washed in PBS and followed by blocking in 10% goat serum (X0907, Dako) in PBS for 30 min at RT. Primary antibody incubation was in 10% goat serum overnight at 4 °C with the following antibodies: αSMA (1:100, ab19671, Abcam), endoglin (1:100, ab69772, Abcam), CD31 (1:100, m0823, Dako), NIK (1:100, sc-8417, Santa Cruz), MECA-79 (1:400, Alexa Fluor 633 conjugated, kindly provided by Eugene Butcher). Isotype control antibodies (concentration matched) were used as negative control on adjacent sections from the same samples. The following day the sections were washed in PBS. Counterstaining was with Alexa Fluor 488, 568, 594 or 647 conjugated goat anti-mouse secondary antibodies (1:500, Invitrogen) and cell nuclei were stained with Hoechst (1:1000 Invitrogen) with 10% goat serum in PBS for 30 min at RT. After PBS washing, sections were mounted with Fluoromount G (ThermoFisher Scientific). CD31^+^/αSMA^+^ vessels with a diameter bigger than 150 µm were classified as large vessels. The whole tissue area was analyzed for the presence of large vessels. The number of large vessels were determined per mm^2^. Vessel distribution was determined by counting different types of blood vessels in two representative images. Two representative images per staining per sample were semi-quantitatively scored (0–3) by 2 assessors while blinded for patients identity and moment of biopsies. Sections were analyzed with a Leica TCS SP8 X mounted on a Leica DMI6000 microscope. Z-stacks were acquired with LAS-X software (version 4.9.0). Adobe Illustrator version 16.0.3 was used for image processing.

### Statistical analysis

A D’Agostino-Pearson omnibus test was used to determine data distribution for normality. SpA and RA patient synovial biopsies were compared with Mann–Whitney U test (non-parametric data) for continuous data or Fisher’s exact test for categorical data. Serum levels and immunofluorescent images between baseline and after 12 weeks treatment were compared using a paired t test (parametric data) or a Wilcoxon matched-pairs signed rank test (non-parametric data). *P* < 0.05 with a 95% confidence interval were considered as statistically significant. The standardized response mean (SRM) was determined by dividing the mean change after treatment for every biomarker by the standard deviation of the change. A SRM < 0.5 indicates a poor potential to identify changes over time, 0.5–0.8 as moderate and > 0.8 as good^27^. All statistics were 2-tailed and performed with GraphPad Prism (GraphPad Software, version 8).

### Ethics approval and consent to participate

All participants gave written informed consent and this study was approved by the local ethics committee of the Amsterdam Medical Center.

## Results

### Anti-IL-17A treatment results in decreased inflammatory, angiogenic and osteogenic serum biomarkers

**Figure 1:**
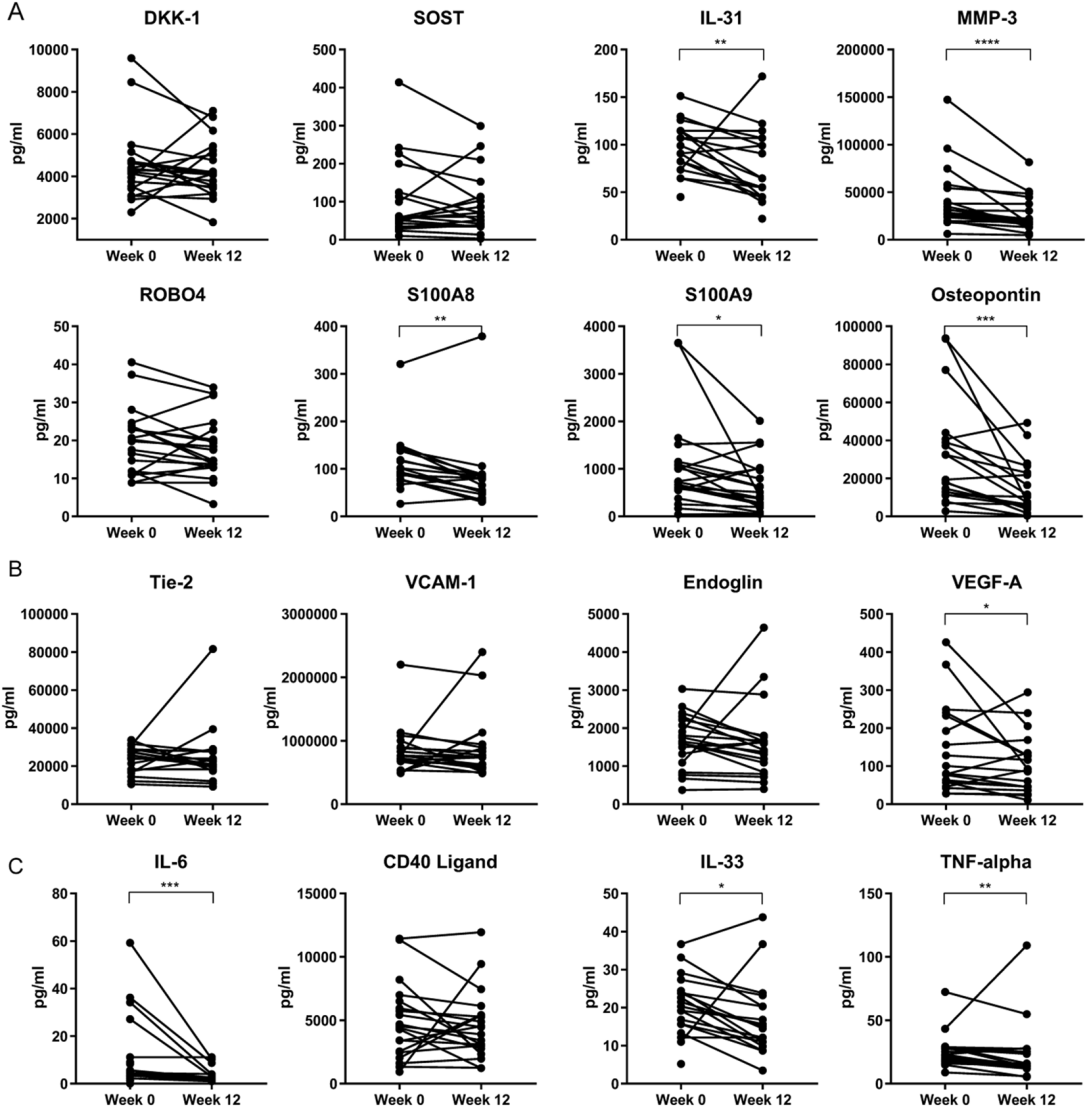
Bone formation, angiogenic and inflammatory markers in serum are downregulated after anti-IL-17A treatment. Effect of anti-IL-17A secukinumab treatment on peripheral blood concentration of tissue remodeling markers (**A**) dickkopf-1 (DKK-1), sclerostin (SOST) interleukin-31 (IL-31), matrix metalloproteinase 3 (MMP-3), roundabout homolog 4 (ROBO4), calcium-binding proteins A8 (S100A8) and A9 (S100A9), osteopontin. Angiogenic markers (**B**) TIE-2, vascular cell adhesion protein 1 (VCAM-1), endoglin (CD105), vascular endothelial growth factor A (VEGF-A). Inflammatory markers (**C**) interleukin-6 (IL-6), CD40 ligand, interleukin-33 (IL-33) and tumour necrosis factor alpha (TNF-α) at week 0 and 12, assessed by luminex technology. Values are paired data for every patient (n = 20). The P value was calculated using a paired t test (IL-31 and ROBO4) or a Wilcoxon matched-pairs signed rank test (DKK-1, SOST, MMP-3, S100A8, S100A9, osteopontin, TIE-2, VCAM-1, endoglin, VEGF-A, IL-6, CD40 ligand, IL-33 and TNF-alpha). *,*P* < 0.05; **,*P* < 0.01; ***,*P* < 0.001; ****,*P* < 0.0001. The graphs were created using GraphPad Prism (version 8, https://www.graphpad.com/) and the figure was created with Adobe Illustrator (version 16.0.3, https://www.adobe.com/products/illustrator.html).

Several inflammatory, angiogenic and osteogenic biomarkers that are increased in active disease could potentially be altered by anti-IL-17A therapy. First, we analyzed baseline serum biomarker concentrations and potential correlations between these factors (suppl. Table 1). CRP and ESR highly correlated (r > 0.75) and had a strong correlation (r > 0.5) with IL-6. Other very strong correlations were observed between MMP-3 and S100A8, IL-31 and TNF-alpha, as well as between S100A8 and IL-31, TNF-alpha and IL-33. VEGF-A was the only angiogenic marker that had a strong correlation with other markers: CRP, IL-31 and S100A8. Next, we assessed whether these biomarkers were affected by 12 weeks of secukinumab treatment in pSpA. Focusing on osteogenic biomarkers in our cohort, BMP-2 levels were often below the detection limit (data not shown), while DKK-1 (*P* = 0.330), SOST (*P* = 0.475) and ROBO4 (*P* = 0.134) did not change. The inflammatory marker IL-31 (*P* = 0.006), which is also associated with structural damage^28^, the tissue remodeling marker MMP-3 (*P* < 0.0001), alarmins S100A8 (*P* = 0.015) and S100A9 (*P* < 0.001), as well as bone remodeling factor osteopontin (*P* < 0.001) significantly decreased in the serum after treatment (Fig. 1A). For the angiogenic serum markers, only VEGF-A (*P* = 0.048) decreased significantly, while TIE-2 (*P* = 0.246), VCAM-1 (*P* = 0.330) and Endoglin (*P* = 0.058) did not change significantly after treatment (Fig. 1B). The % of change in VEGF correlated significantly with the % of change in BASDAI (r = 0.51, *P* = 0.02). Inflammatory markers IL-6 (*P* = 0.001), IL-33 (*P* = 0.017) and TNF-α (*P* = 0.005) decreased significantly, while CD40L (*P* = 0.2774) remained unaltered (Fig. 1C). Concerning correlations of baseline serum levels with clinical parameters that were published earlier^13^, the DAS28 (ESR) at week 12 correlated with baseline ESR (r = 0.538, *P* = 0.014) and S100A9 (r = 0.617, *P* = 0.004). The BASDAI change only correlated with baseline CRP (r = -0.699, *P* < 0.001), ESR (r = − 0.451, *P* = 0.046) and IL-6 (r = − 0.482, *P* = 0.037). Next, we analyzed the potential of the biomarkers to change due to treatment by calculating the SRM, which shows the capability to identify changes over time^27^. The SRM for MMP-3 (− 0.85) and S100A8 (− 0.86) was good after anti-IL-17A treatment, whereas SRM was moderate for ESR (− 0.63), IL-31 (− 0.52), S100A9 (− 0.51), osteopontin (− 0.77), IL-6 (− 0.68) and IL-33 (− 0.57) (Table 1).

**Table 1:** Potential to change after secukinumab in all, CRP-low and CRP-high patients.

	Total (n = 20)	CRP-low (n = 12)	CRP-high (n = 8)
CRP	− 0.49	− 0.05	** − 0.98**
ESR	* − 0.63*	− 0.02	** − 1.74**
DKK-1	− 0.11	− 0.33	0.18
IL-6	* − 0.68*	** − 1.33**	** − 1.22**
IL-31	* − 0.52*	**− 1.21**	− 0.21
MMP-3	** − 0.85**	**− 1.58**	** − 1.11**
ROBO4	− 0.33	− 0.47	− 0.17
S100A9	* − 0.51*	− 0.49	* − 0.73*
Tie-2	0.08	0.11	0.03
VCAM-1	0.14	− 0.38	*0.52*
CD40 Ligand	− 0.11	− 0.44	0.11
Endoglin	− 0.04	− 0.27	0.24
IL-33	* − 0.57*	** − 1.17**	− 0.22
Osteopontin	* − 0.77*	** − 0.95**	* − 0.73*
S100A8	** − 0.86**	*− 0.55*	** − 1.71**
SOST	− 0.14	− 0.33	0.04
TNF-alpha	− 0.13	0.00	* − 0.77*
VEGF-A	− 0.40	− 0.29	* − 0.62*

Emphasis mark the potential to change; > 0.8, good (bold); > 0.5, moderate (italics). *SRM* standardized response mean; *DKK-1* dickkopf-1; SOST sclerostin; *IL-31* interleukin-31; *MMP-3* matrix metalloproteinase 3; *ROBO4* roundabout homolog 4; *S100A8* and *S100A9* calcium-binding proteins; osteopontin. TIE-2; *VCAM-1* vascular cell adhesion protein 1; endoglin; *VEGF-A* vascular endothelial growth factor A; *CRP* C-reactive protein, cutoff level 5 mg/L; *ESR* erythrocyte sedimentation rate; *IL-6* interleukin-6; CD40 ligand; *IL-33* interleukin-33 and *TNF-α* tumour necrosis factor alpha.

### Potential biomarkers in CRP-low patients

CRP is not very specific for SpA and there was only a strong correlation with a few markers. We therefore analyzed the subgroup with low baseline CRP levels (< 5 mg/L, the threshold in our laboratory). In the 12 patients with low CRP levels, IL-31 (*P* = 0.004), MMP-3 (*P* < 0.001), osteopontin (*P* = 0.003), VCAM-1 (*P* = 0.034), endoglin (*P* = 0.043), IL-33 (*P* = 0.039) and TNF-alpha (*P* = 0.034) decreased significantly after treatment. We determined whether these markers might be of added value in CRP-low patients by calculating the potential to change (Table 1). In the CRP-low group, IL-31, MMP-3, osteopontin IL-6, and IL-33 had a good SRM. In the CRP-high group (> 5 mg/L), MMP-3, S100A8, CRP, ESR and IL-6 had a good SRM. Our data indicate that in CRP-low SpA patients treated with secukinumab, the markers IL-31, MMP-3, osteopontin and IL-33 decreased significantly and were most sensitive to change.

### SpA ST is characterized by an increase in large blood vessels compared to RA

**Figure 2:**
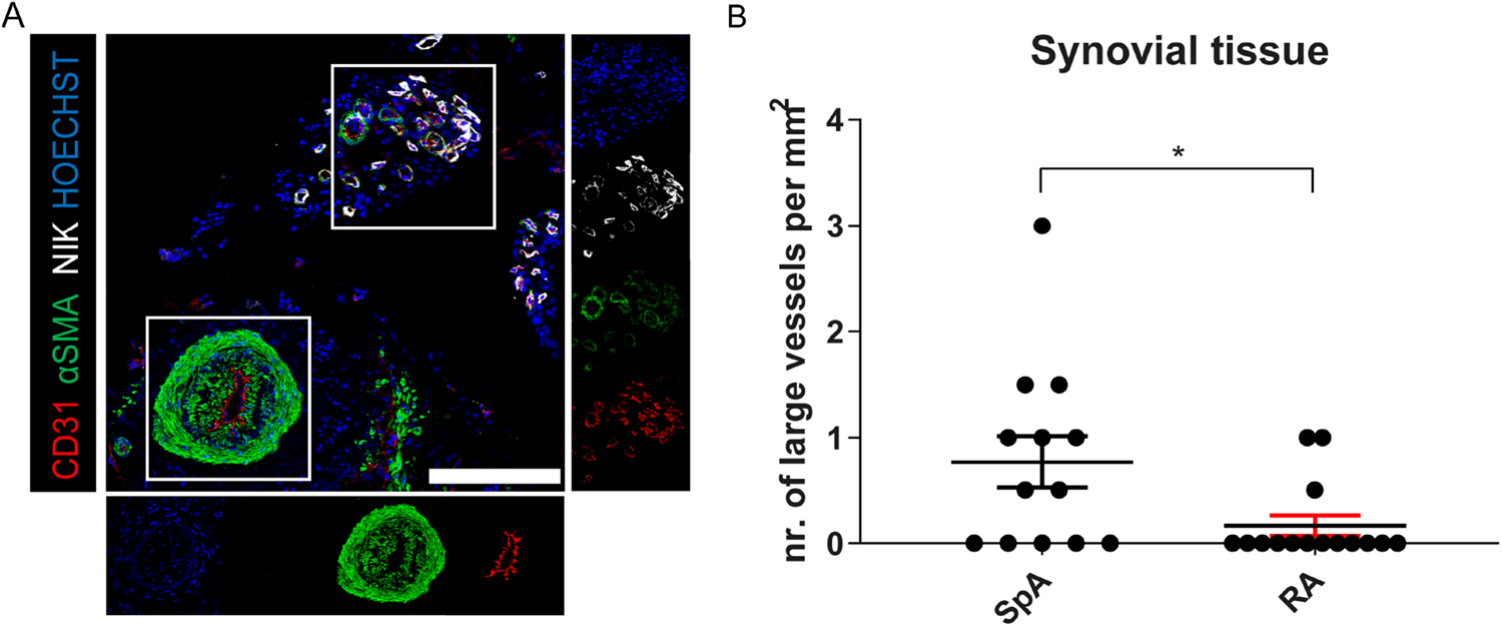
The presence of large vessels in inflamed ST. (**A**) An immunofluorescent image of a SpA patient showing a large vessel next to a group of smaller vessels in spondyloarthritic ST. Scalebar 200 µm. (**B**) Amount of large vessels per mm^2^ in SpA versus rheumatoid arthritis (n = 13 SpA, n = 15 RA). The P value was calculated using a Mann–Whitney test. Values are represented as mean ± SEM. *,*P* < 0.05. The image was made with LAS-X software (version 4.9.0, https://www.leica-microsystems.com/products/microscope-software/p/leica-las-x-ls/), the graph was created using GraphPad Prism (version 8, https://www.graphpad.com/) and the figure was created with Adobe Illustrator (version 16.0.3, https://www.adobe.com/products/illustrator.html).

Synovial angiogenesis contributes substantially to persistence of inflammation^29^. In order to compare angiogenesis of ST in SpA versus RA, we analyzed synovial biopsies from 13 SpA and 15 RA patients with active disease. Patient characteristics are listed in suppl.Table 2. To evaluate whether angiogenesis and non-canonical NF-κB signaling in particular might be important for bone formation and thus different between SpA and RA, we analyzed synovial cryosections with IF for vascular markers CD31 (pan-endothelial marker), αSMA (pericyte marker) and NF-κB Inducing Kinase (NIK) (immature vessels) to assess vessel distribution and size (Fig. 2A). With these markers we could distinguish between immature (CD31^+^/NIK^+^/αSMA^-^), mature (CD31^+^/NIK^+/-^/αSMA^+^) and large vessels (CD31^+^/NIK^-^/αSMA^+^ with a diameter of at least 150 µm). The number of immature vessels and mature vessels per field of view were comparable between SpA and RA. IF staining revealed that large vessels were present in the synovium of 7/13 (54%) SpA patients and 3/15 (20%) RA patients (*P* = 0.114). The total amount of large vessels was significantly higher in SpA compared to RA ST (*P* = 0.0201) (Fig. 2B). In line with previous results^21^, NIK positivity was mainly observed in small vessels. Next, we stained for CD31, αSMA and endoglin as a marker for activated endothelial cells that are associated with angiogenesis and bone formation to assess whether there were differences in endoglin associated vessels between SpA and RA (suppl. Fig. 1)^8^. The distribution of immature, active and mature vessels was equal between SpA and RA (suppl. Fig. 1B). Most vessels (80% in both SpA and RA) were positive for endoglin, indicating that the majority of ECs in these blood vessels are activated.

### ST high endothelial venules and lymphoid aggregates diminish after anti-IL-17A treatment

**Figure 3:**
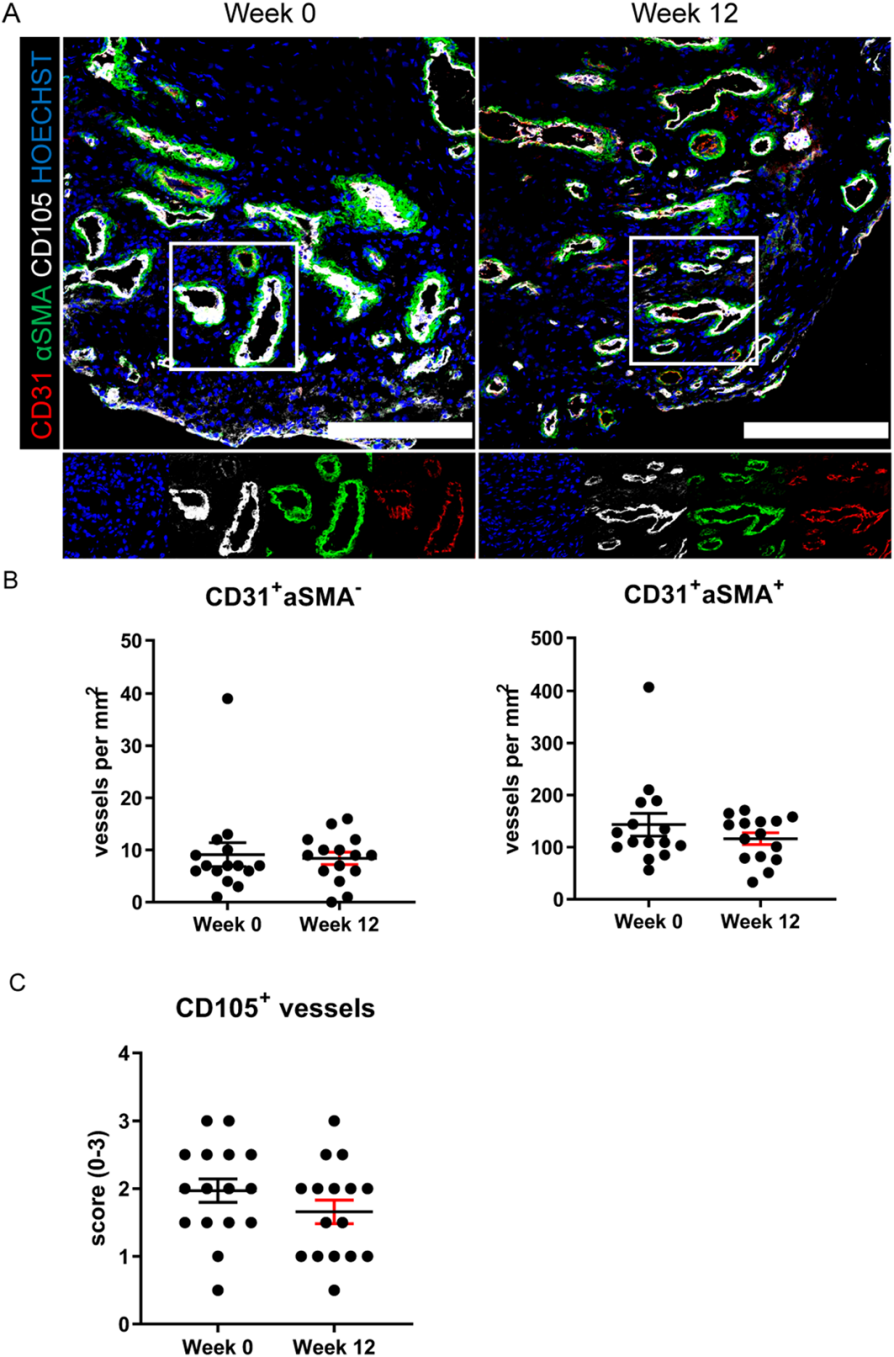
Conventional angiogenic markers are not affected after 12 weeks of anti-IL17A. (**A**) Representative paired images of synovial biopsies obtained at week 0 and after 12 weeks of treatment. Scale bars are 200 µm (n = 15). (**B**) Amount of immature (CD31^+^/αSMA^-^) and mature (CD31^+^/αSMA^+^) vessels per mm^2^. (**C**) Semi-quantitative score for presence of CD105^+^ vessels. The P value was calculated using a Wilcoxon matched-pairs signed rank test (B and C). Values are represented as mean ± SEM. The images were made with LAS-X software (version 4.9.0, https://www.leica-microsystems.com/products/microscope-software/p/leica-las-x-ls/), the graphs were created using GraphPad Prism (version 8, https://www.graphpad.com/) and the figure was created with Adobe Illustrator (version 16.0.3, https://www.adobe.com/products/illustrator.html).

Besides assessing systemic effects of secukinumab treatment in serum, we also analyzed ST biopsies taken before and after 12 weeks of treatment. To determine whether angiogenesis was affected by anti-IL-17A therapy, we stained synovial sections before and after treatment for CD31, αSMA and endoglin (Fig. 3A). Conventional angiogenic markers were unaffected by secukinumab treatment as the density of CD31^+^/αSMA^-^ immature (*P* = 0.659) and CD31^+^/αSMA^+^ mature (*P* = 0.762) vessels did not change after treatment in the ST based on the total number of vessels (Fig. 3B). There was also no change in the number of large vessels before and after treatment (data not shown). Also endoglin expression, which is associated with bone formation was unaffected in the synovium after treatment (*P* = 0.201) (Fig. 3C).

**Figure 4:**
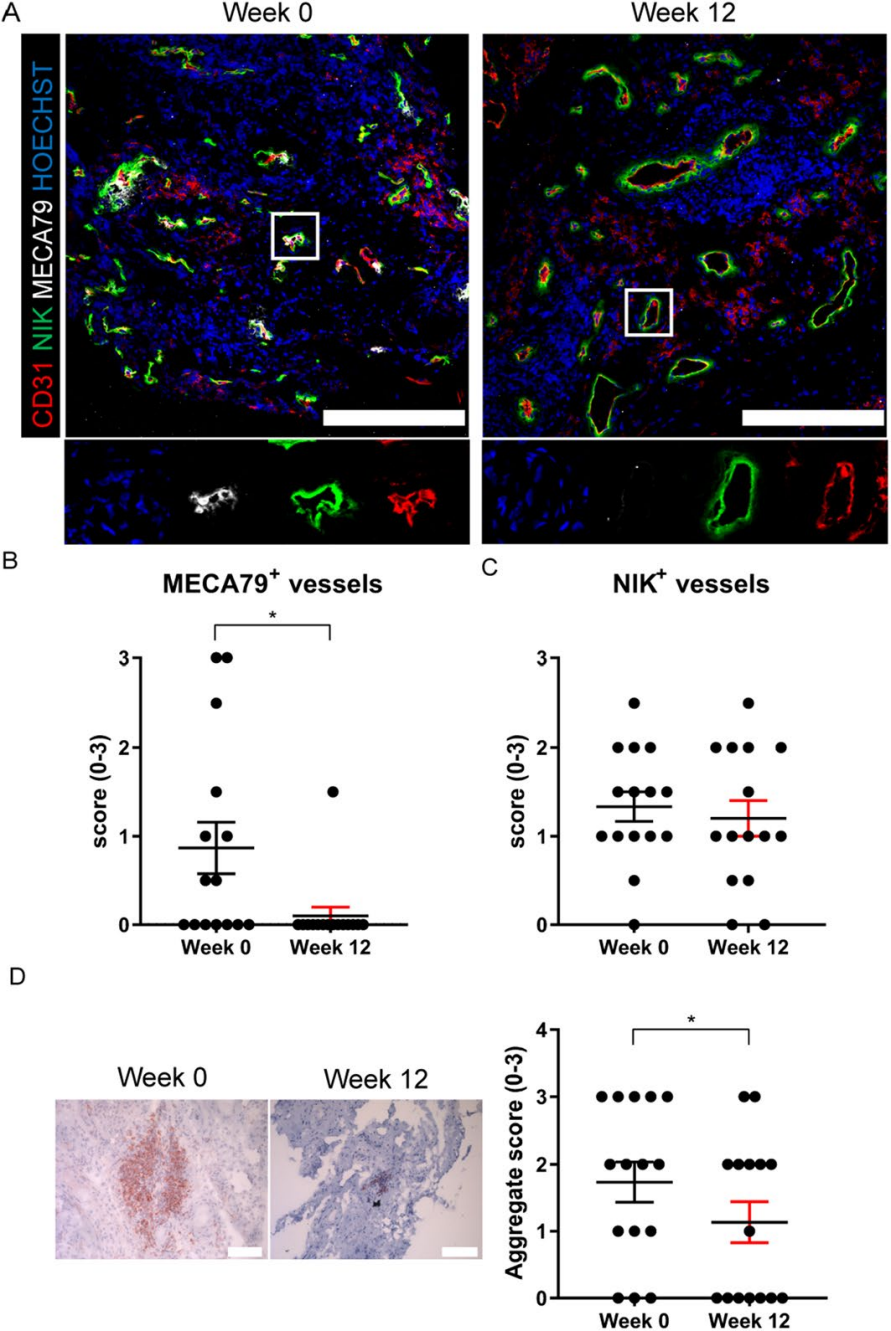
High endothelial venules in ST diminish after anti-IL-17A treatment. (**A**) Representative paired images of synovial biopsies obtained at week 0 and after 12 weeks of treatment. Scalebars 200 µm (n = 15). (**B**) Semi-quantitative score for presence of MECA79^+^ vessels. (**C**) Semi-quantitative score for presence of NIK^+^ vessels. (**D**) Paired CD20 stained cryosections. Semi-quantitative scoring for aggregates. Scalebars 100 µm (n = 17). The P value was calculated using a paired t test (C and D) or a Wilcoxon matched-pairs signed rank test (B). Values are represented as mean ± SEM. *,*P* < 0.05. The images were made with LAS-X software (version 4.9.0, https://www.leica-microsystems.com/products/microscope-software/p/leica-las-x-ls/), the graphs were created using GraphPad Prism (version 8, https://www.graphpad.com/) and the figure was created with Adobe Illustrator (version 16.0.3, https://www.adobe.com/products/illustrator.html).

To assess if the anti-IL-17A treatment had an effect on other specialized blood vessels and lymphoid aggregates in the ST, we performed IF staining of tissue sections for CD31, NIK and MECA79 to identify HEVs (Fig. 4A). Quantification of IF stainings confirmed that the number of MECA79^+^ blood vessels decreased significantly after treatment (*P* = 0.031), while the total number of NIK^+^ blood vessels (*P* = 0.677) did not change significantly (Fig. 4B,C). To evaluate if the decrease in HEVs had an effect on ELS, we assessed the presence of these structures in the ST. After secukinumab treatment, there was a significant decrease in ELS size (*P* = 0.027) (Fig. 4D). These data indicate that secukinumab treatment results in a specific reduction of HEVs which is accompanied by less ELS in the ST.

## Discussion

In the current study, we assessed various markers of angiogenesis and tissue remodeling in serum and inflamed synovium focusing on the effects of secukinumab treatment in pSpA. In the first part of this study, we focused on the question whether serum levels of soluble factors involved in inflammation, angiogenesis and tissue remodeling/new bone formation change in SpA patients after 12 weeks of anti-IL-17A treatment. DKK-1 and SOST (gene regulating sclerostin) act as negative regulators of bone formation through Wnt signaling and may predict structural damage progression in axial SpA^30^. Interestingly, SOST immune complexes have been found in SpA^31^. Studies reporting DKK-1 and SOST levels in SpA patients have been conflicting and TNF inhibition does not seem to alter DKK-1 in SpA^23,32^, while it might increase SOST levels^32^. We also did not observe changes in DKK-1 and SOST levels after IL-17A blockade. Similar to our study, a short course of secukinumab treatment in PsA patients did not influence the levels of these markers^33^. However, this does not exclude that these markers may change after longer periods of treatment. For DKK-1 this could be due to the fact that total protein levels were measured instead of only receptor bound DKK-1^30^. Alternatively, the unaltered levels may be due to concurrent arthritis-associated bone degradation with erosions that is linked to increased levels of DKK-1 and SOST, which is also an integral part of SpA^34^. IL-31 is part of the IL-6 family of cytokines and a preliminary study suggests that high baseline IL-31 levels are not only associated with reduced new bone formation based on the modified Stoke Ankylosing Spondylitis Spinal Score (mSASSS) in early SpA, but also with low bone mineral density^28^. We observed that IL-31 levels decreased significantly after treatment, but whether this decrease was merely the result of reduced inflammation or also has implications regarding bone remodeling remains to be determined. Interestingly, IL-17 has been shown to induce the inflammation and tissue remodeling factors S100A8 and S100A9, thereby possibly providing resistance to anti-angiogenic treatment^35^. S100A8 and S100A9 (as heterodimer called calprotectin) were previously proposed by us and others as serum biomarkers for clinical response in SpA after anti-TNF treatment^12,27^. Similarly, we observed that S100A8 and S100A9 decreased after IL-17A blockade, suggesting that these factors may be good biomarkers for clinical treatment response. The patient with IBD in clinical remission did have one of the highest S100A8 and S100A9 baseline levels, which may fit with elevated calprotectin levels found in SpA patients at risk of bowel inflammation and in IBD patients^36^. Nonetheless, both S100A8 and S100A9 declined after secukinumab treatment in this patient and no flare of IBD was recorded^37^. MMPs are involved in the breakdown of extracellular matrix proteins and are pivotal for tissue remodeling. MMP production is induced by IL-17 and MMP-3 has been demonstrated to be a biomarker for effective treatment in SpA^15,38^. In line with this, MMP-3 decreased significantly upon anti-IL-17A treatment with a strong SRM, in particular in CRP-low patients. Osteopontin, a late marker of bone formation, is elevated in SpA compared to healthy controls, but did not decrease after TNF inhibition^39^. We observed a significant decrease in osteopontin, which may indicate that osteopontin expression may be IL-17A dependent^40^ and deserves new attention. An endothelial-osteoblast link through the ROBO/SLIT pathway is associated with bone formation in mice^41^. The same pathway may contribute to EC dysfunction and microangiopathy in systemic sclerosis, which is also characterized by tissue remodeling^42^. Therefore, we measured ROBO4 in the serum as a potential biomarker for tissue remodeling, but this did not change after anti-IL-17A treatment. To summarize, we are the first to describe the effects of secukinumab treatment on tissue remodeling biomarkers in the serum of SpA patients. MMP-3 and especially osteopontin seem to be biomarkers responsive to secukinumab treatment. Notwithstanding, it must be noted that it is still unknown how serum levels correlate to local bone remodeling and thus if these biomarkers may be of predictive value to structural changes in SpA.

Although angiogenesis and bone formation are closely linked processes both involved in the pathogenesis of SpA, only the angiogenic marker VEGF has been studied extensively in serum. Elevated serum levels of VEGF may predict radiographic progression in the spine^5^. However, although VEGF levels decrease after TNF inhibition, VEGF did not predict new bone formation in anti-TNF treated SpA patients^43^. The effects of anti-IL-17A on serum angiogenic markers such as VEGF were hitherto unknown. VEGF-A declined significantly after secukinumab treatment, which fits with the fact that IL-17A has pleiotropic effects on EC, including upregulation of adhesion molecules and stimulation of angiogenesis^15^. TIE-2 and VCAM-1 are associated with active RA^44,45^, but data in SpA are limited to observations in different tissues without intervention^44,46^. TIE-2 may be less important as a biomarker in SpA, since expression was much less in SpA synovium compared to RA^44^. In our study TIE-2, VCAM-1 and endoglin did not change significantly.

We also evaluated several inflammatory cytokines that have been associated with disease activity in SpA. TNF, a pivotal cytokine in the pathogenesis of SpA decreased significantly after anti-IL-17A treatment. IL-6 is one of the most studied biomarkers in SpA^23^ and we observed a clear decrease in serum IL-6 levels. Since IL-6 and TNF can have opposing effects on DKK-1 expression^47^, this may explain our unaltered DKK-1 levels. CD40L, which has been demonstrated to be elevated in early AS patients^28^ did not decrease after secukinumab treatment. It has been recently highlighted that IL-33 secretion by endoglin^+^ EC is important for bone formation^8^. IL-33 decreased significantly after secukinumab treatment. This fits with other studies correlating IL-33 with disease activity^23^. The observed effect is likely to be specific for secukinumab treatment, since IL-17 and IL-33 expression are closely linked, most notably during infections and in allergy^48,49^.

Tissue remodeling is a long, slow process, which is challenging to predict or monitor. Given the importance of angiogenesis in tissue remodeling, we focused in the second part on tissue angiogenesis. Since samples of tissues that undergo remodeling (i.e. bony spurs at tendon insertions, ankylosed joints or spine) are difficult to obtain in SpA, we examined the inflamed ST of peripheral joints^50,51^. Several studies report that ST of SpA patients may have more vascularity compared to RA^3,4^. However, we found no apparent difference in vascularity in our cohort. Nevertheless, we did observe that active pSpA patients (including PsA) have more large synovial blood vessels than RA patients, which could be a marker for longstanding angiogenic activity or maturation of blood vessels. Since blood vessel diameter is correlated with blood flow, it is an important parameter for perfusion of the tissue^52^. Our results iterate that ST in SpA may indeed have increased vascularization and blood flow compared to RA. Endoglin^+^ (CD105) EC are regarded as angiogenic and are also associated with new bone formation^8^. Given that SpA is characterized by pathological bone formation, which is absent in RA, we investigated potential differences in endoglin^+^ blood vessels in ST. In this cohort we did not observe a difference between RA and SpA, which could mean that endoglin is not higher expressed in SpA and that these blood vessels do not contribute to new bone formation. However, endoglin expression in blood vessels might only be specifically elevated at the site of new bone formation in SpA, but as already indicated these tissues are very difficult to obtain in patients.

Next, we assessed angiogenic changes after secukinumab treatment. Inflamed synovium is full of immature blood vessels and correlates with progressive disease, while these vessels are virtually nonexistent in healthy synovium^53^. In the original study a clear reduction in synovial inflammation was shown, while the number of vWF^+^ blood vessels did not change significantly^13^. Notwithstanding, specific subsets of blood vessels may still decline substantially after treatment with anti-IL-17A^15,53^. Therefore, we stratified for immature CD31^+^/αSMA^-^ vs. mature CD31^+^/αSMA^+^ vessels, but did not observe a change based on the synovial stainings. Since IL-17A is important for the formation of ELS in arthritis^17^, we analyzed ST for the presence of MECA79^+^ HEVs, specialized blood vessels that facilitate migration of immune cells into lymph nodes and inflamed tissues. Interestingly, HEVs decreased significantly after secukinumab treatment, accompanied by a reduction in ELS. Of note, it may take longer than the duration of 12 weeks for all ELS to disappear. Earlier research has shown that ELS in RA and SpA are very much comparable^54,55^. We did not observe a difference in the number of NIK^+^ vessels, implying that non-canonical NF-κB signaling is unaltered and does not depend on IL-17A in SpA. This fits with the fact that serum levels of sCD40L, which can induce non-canonical NF-κB signaling^21^, were also not altered.

Our study has several limitations. First, a sample size of 20 is relatively small and a larger sample size is needed to perform subgroup analysis. Also, due to the study design, we did not have a placebo group. These data are similar to studies using TNF inhibition, but comparisons are difficult as study designs differ and head-to-head comparisons have not been done^23,27,55^. Nevertheless, MMP-3^27^ and osteopontin^39^ seem to decrease more after anti-IL-17A treatment than after TNF inhibition, and especially in CRP-low patients have a good SRM. Whether anti-IL17 treatment could have more impact on tissue remodeling/new bone formation than anti-TNF therapies remains unknown and warrants (long-term) head-to-head comparison. Interestingly, while HEVs and ELS in ST of PsA and pSpA patients did not decrease significantly after TNF inhibition^55,56^, it did after secukinumab treatment in this study. This could also help to explain why some patients respond better to one or the other treatment.

In conclusion, this is the first study to assess biomarkers of angiogenesis and tissue remodeling after secukinumab treatment in SpA. We demonstrate that serum levels of IL-31, MMP-3, S100A8, S100A9, osteopontin, VEGF-A, IL-6, IL-33 and TNF-α declined significantly after secukinumab treatment, confirming that anti-IL-17 has pleiotropic, beneficial effects on inflammation, angiogenesis and tissue remodeling. In addition, we observed a decrease in synovial HEVs and ELS. These results demonstrate that anti-IL-17 treatment impacts on different pathological processes involved in SpA and highlight the possibility of identifying biomarkers in serum and/or ST to monitor these processes. After formal validation of MMP-3, S100A8, S100A9 and/or osteopontin as markers of tissue remodeling and treatment response in SpA, analysis of serum samples before and soon after starting a new treatment may indicate whether the initiated treatment is likely to be effective over time or needs to be changed. To prove this, further research needs to be done, including confirmation of our findings in larger studies and correlation with radiographic data.

## Supplementary information

Supplementary Information 1.

## Data Availability

The datasets during and/or analyzed during the current study available from the corresponding author on reasonable request.
