# Supplementary material for: Anti-IL-17A treatment reduces serum inflammatory, angiogenic and tissue remodeling biomarkers accompanied by less synovial high endothelial venules in peripheral spondyloarthritis
Source: Sci Rep. 2020 Dec 3;10:21094. doi: 10.1038/s41598-020-78204-6 (PMC7713433; doi:10.1038/s41598-020-78204-6)
Supplement: Supplementary file 1 — Supplementary Information 1. [file 41598_2020_78204_MOESM1_ESM.pdf]

Anti-IL-17A treatment reduces serum inflammatory, angiogenic and tissue remodeling biomarkers accompanied by less synovial high endothelial venules in peripheral spondyloarthritis

Merlijn H. Kaaij[1,2], Boy Helder[1,2], Leonieke J.J. van Mens[1], Marleen G.H. van de Sande[1], Dominique L.P. Baeten[1,2], and Sander W. Tas[1,2]

[1]Amsterdam UMC, University of Amsterdam, Department of Rheumatology and Clinical Immunology, Amsterdam Rheumatology and immunology Center, Meibergdreef 9, Amsterdam, Netherlands, [2]Amsterdam UMC, University of Amsterdam, Department of Experimental Immunology, Amsterdam Infection and Immunity Institute, Meibergdreef 9, Amsterdam, Netherlands

	CRP	ESR	DKK-1	SOST	IL-31	MMP-3	ROBO4	S100A8	S100A9	OPN	TIE-2	VCAM-1	Endoglin	VEGF-A	IL-6	CD40L	IL-33
ESR	0,833****																
DKK-1	-0,018	0,032															
SOST	0,408	-0,144	0,417														
IL-31	0,393	0,361	0,438	0,320													
MMP-3	0,481*	0,333	0,179	0,457*	0,796****												
ROBO4	0,034	0,006	0,310	0,690***	0,384	0,606**											
S100A8	0,408	0,364	0,267	0,222	0,924****	0,777****	0,217										
S100A9	0,553*	0,594**	0,230	-0,044	0,462*	0,101	-0,090	0,449									
OPN	0,082	0,041	-0,119	0,193	0,069	0,298	0,193	0,012	-0,046								
TIE-2	0,078	0,063	0,011	-0,063	0,087	0,266	0,350	0,187	0,125	0,183							
VCAM-1	-0,278	-0,134	0,377	-0,168	-0,140	-0,265	-0,194	-0,247	-0,280	0,165	-0,218						
Endoglin	-0,284	-0,261	0,177	0,221	0,191	0,064	0,214	0,074	-0,211	0,214	-0,258	0,272					
VEGF-A	0,577**	0,398	0,321	0,085	0,518*	0,373	-0,125	0,551*	0,444*	0,066	-0,178	0,213	-0,037				
IL-6	0,706***	0,537*	-0,119	0,115	0,457	0,636**	0,185	0,669**	0,449	0,048	0,428	-0,526*	-0,314	0,459			
CD40L	-0,011	0,029	0,620**	0,057	0,204	-0,081	-0,125	0,135	0,344	-0,328	-0,146	0,159	-0,263	0,395	-0,116		
IL-33	0,288	-0,021	0,102	0,342	0,743***	0,676**	0,315	0,761***	0,131	-0,015	0,227	-0,404	0,197	0,420	0,589**	0,053	
TNF-alpha	0,427	0,260	0,142	0,183	0,726***	0,781****	0,324	0,800****	0,214	-0,159	0,203	-0,314	0,030	0,432	0,730***	0,085	0,700***

Supplemental table 1. Baseline correlations of bone formation, angiogenic and inflammatory markers. Spearman's correlation coefficients are shown for all markers in pSpA patients at baseline. Osteopontin (OPN), CD40 ligand (CD40L). Colors mark the strength of correlation; >0.75, very strong (dark green); >0.5, strong (light green); >0.25, moderate (yellow); <0.25, moderate (light blue); <0.5, (blue). *, P < 0.05; **, P < 0.01; ***, P < 0.001; ****, P < 0.0001.

Variables	SpA (n=13)	RA (n=15)
Male/female	9/4	5/10
Age, median (IQR) years	44 (40-55.5)	56 (51-58)
Disease duration, median (IQR) months	41 (0.6-115)	6.4 (4.0-8.5)
Axial disease, n	1 (7.7%)	NA
Uveitis, n	0 (0%)	0 (0%)
IBD, n	0 (0%)	0 (0%)
Rheumatoid factor+, n	0 (0.00%)	7 (46.7%)
ACPA+, n	0 (0.00%)	4 (26.7%)
66 Swollen joint count, median (IQR)	5 (1-5)	4 (1-11)
68 Tender joint count, median (IQR)	5 (1.5-12.5)	4 (1-12)
DAS28, median (IQR)	4.77 (4.25-5.7)	4.22 (3-6.3)
ESR, median (IQR) mm/hour	21 (9-40.5)	15 (7-35)
CRP, median (IQR) mg/L	10.5 (3.5-24.96)	3.4 (3-28.3)
NSAIDs	3 (23%)	10 (67%)
Corticosteroids	1 (8%)	3 (20%)
DMARDs	4 (31%)	8 (53%)

Supplemental table 2. Patient characteristics SpA and RA cohort. *IQR* interquartile range; *IBD* Inflammatory Bowel Disease; *ACPA* anti-citrullinated protein antibodies; *NSAIDs*, nonsteroidal anti- inflammatory drugs; *DMARDs*, disease-modifying anti-rheumatic drugs.

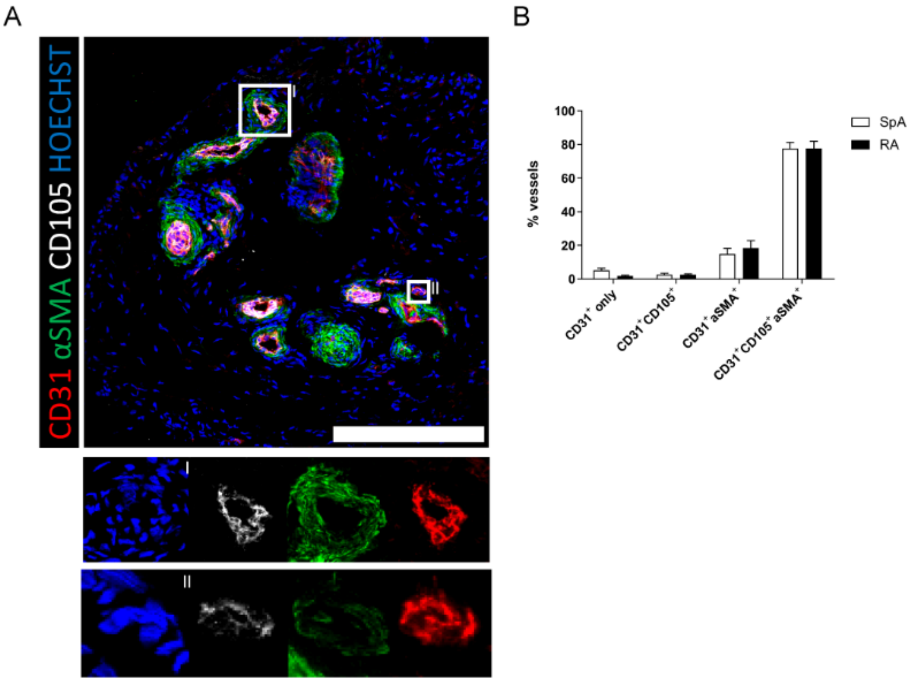

Supplemental figure 1. The distribution of vessels in inflamed ST. A An image showing different vessels in spondyloarthritic ST. Scalebar 200 µm. I: CD31^{+}, CD105^{+}, alphaSMA^{+} vessel. II: CD31^{+}, CD105^{low}, alphaSMA^{-} vessel. **B** Quantification for vessel maturity in SpA versus rheumatoid arthritis (n = 14 SpA, n = 16 RA). Values are represented as mean ± SEM. The image were made with LAS-X software (version 4.9.0, https://www.leica-microsystems.com/products/microscope-software/p/leica-las-x-ls/), the graph was created using GraphPad Prism (version 8, https://www.graphpad.com/) and the figure was created with Adobe Illustrator (version 16.0.3, https://www.adobe.com/products/illustrator.html).
